# Ultrasonographic detection and assessment of the severity of Crohn's disease recurrence after ileal resection

**DOI:** 10.1186/1471-230X-10-69

**Published:** 2010-07-01

**Authors:** Nadia Pallotta, Maurizio Giovannone, Patrizio Pezzotti, Alessandro Gigliozzi, Fausto Barberani, Daria Piacentino, Naima Abdulkadir Hassan, Giuseppina Vincoli, Mauro Tosoni, Alfredo Covotta, Adriana Marcheggiano, Mauro Di Camillo, Enrico Corazziari

**Affiliations:** 1 Department of Scienze Cliniche, University of Rome "Sapienza", Policlinico "Umberto I", V.le del Policlinico 155, Rome 00161, Italy; 2 Gastroenterology Unit, "S. Camillo De Lellis" Hospital, V.le Matteucci 9, Rieti 02100, Italy; 3 Lazio Sanità - Agenzia di Sanità Pubblica, Via di S. Costanza 53, Rome 00198, Italy; 4 Department of Scienze Chirurgiche e Tecnologiche Mediche Applicate, University of Rome "Sapienza", Policlinico "Umberto I", V.le del Policlinico 155, 00161 Rome, Italy

## Abstract

**Background:**

Recurrence and severity of Crohn's disease mucosal lesions after "curative" ileal resection is assessed at endoscopy. Intramural lesions can be detected as increased wall thickness at Small Intestine Contrast Ultrasonography (SICUS).

Aims. To assess after ileal resection whether: 1) SICUS detects recurrence of Crohn's disease lesions, 2) the intestinal wall thickness measured at the level of ileo-colonic anastomosis predicts the severity of endoscopic lesions, 3) the extension of intramural lesions of the neo-terminal ileum is useful for grading severity of the recurrence, 4) the combined measures of wall thickness of the ileo-colonic anastomosis and of the extension of intramural lesions at level of the neo-terminal ileum may predict the endoscopic Rutgeerts score

**Methods:**

Fifty eight Crohn's disease patients (M 37, age range 19-75 yrs) were prospectively submitted at 6-12 months intervals after surgery to endoscopy and SICUS for a total of 111 observations.

**Results:**

Six months or more after surgery wall thickness of ileo-colonic anastomosis > 3.5 mm identified 100% of patients with endoscopic lesions (p < 0.0001). ROC curve analysis, combining wall thickness of ileo-colonic anastomosis and the extension of intramural lesions of neo-terminal ileum, discriminated (0.95) patients with, from those without, endoscopic lesions. Performing two multiple logistic regression analyses only wall thickness of ileo-colonic anastomosis and extension of neo-terminal ileum intramural lesions were significantly associated with absence or presence of endoscopic lesions. An ordinal polychotomus logistic model, considering all investigated variables, confirmed that only SICUS variables were associated with endoscopic grading of severity.

**Conclusions:**

In patients submitted to ileal resection for Crohn's disease non-invasive Small Intestine Contrast Ultrasonography 1) by assessing thickness of ileo-colonic anastomosis accurately detects initial, minimal Crohn's disease recurrence, and 2) by assessing both thickness of ileo-colonic anastomosis and extension of intramural lesions of neo-terminal ileum grades the severity of the post-surgical recurrence.

## Background

In patients submitted to surgery for ileo-colonic Crohn's disease (CD), recurrence of CD intestinal lesions at the level of ileo-colonic anastomosis and neo-terminal ileum is extremely common. Indeed, it is now firmly established that surgery, even though apparently radical and despite initial clinical remission, does not offer a definitive cure. So far the natural course of initial, minimal postoperative lesions at the level of neo-terminal ileum has been assessed with ileo-colonoscopy and a seminal prospective endoscopic cohort study demonstrated that the severity of postoperative recurrent CD lesions is the best predictor of the clinical outcome [1]. Endoscopy, however, is an invasive procedure that reduces the patient's compliance to regular follow-up controls preventing the detection of early and low grade recurrent lesions [2,3]. Furthermore, the intramural nature of Crohn's disease intestinal lesions cannot be assessed with endoscopy. Small intestine contrast ultrasonography (SICUS) performed after the ingestion of oral contrast makes possible to measure the wall thickness and the luminal diameter of the small bowel [4,5]. In Crohn's disease of the small bowel intramural lesions cause an increased thickness of the intestinal wall that can be accurately detected with the non invasive SICUS [6,7]. In patients submitted to ileal resection, previous oral contrast ultrasound studies [8,9], assessing the wall thickness of the neo-terminal ileum, showed a low sensitivity in detecting initial, low grade CD recurrence (i.e. Rutgeerts score 1 and 2) that may be limited only to the ileo-colonic anastomosis [1]. Therefore the aims of the present study were to assess in CD patients submitted to ileal resection whether

1. a non invasive technique such as SICUS can be used to detect minimal CD lesions, i.e. low grade recurrence limited to ileo-colonic anastomosis

2. the intestinal wall thickness measured at the level of ileo-colonic anastomosis can predict the severity of endoscopic lesions according to Rutgeerts score

3. the evaluation of the extension of intramural lesions at the level of the neo-terminal ileum may be useful for grading the severity of the recurrence

4. the combined measures of wall thickness of the ileo-colonic anastomosis and the extension of wall thickening of the neo-terminal ileum may predict the endoscopic Rutgeerts score

## Methods

### Patients

As a part of a long-term prospective follow up study 58 consecutive patients with Crohn's disease of the small bowel, submitted to resection of macroscopically diseased bowel, were evaluated since the year 2000. Patients underwent surgery because of the following conditions; 32 (55%) for symptomatic obstructive ileal strictures, 8 (14%) for abscess and fistulae, 2 (3%) for perforation, 15 (26%) for strictures and fistulae/abscess and 1 unresponsive to medical treatment.

Medical history, including abdominal and extra-abdominal complaints, associated disease, CD behaviour, smoking status at diagnosis, CDAI at referral, family history, location of CD, duration of the disease before surgery, number of surgical resections, post-operative medical treatment and pathological studies of the resection specimen and of the section margins were assessed.

Informed consent was obtained from each subject and the study protocol was approved by the local ethics committee of our University Hospital # 918.

### Protocol of the study

Patients were prospectively evaluated with SICUS and ileo-colonoscopy with scheduled follow-up at 6 months and 1 year after surgery and at regular intervals of 6-12 months thereafter. Each patient was initially submitted to a standardized clinical interview and a physical examination performed by certified and experienced gastroenterologists. Patients were then consecutively submitted to biochemistry, SICUS and within 2 weeks interval on different days and in random order, to a ileo-colonoscopy. When deemed necessary additional investigations, including upper GI endoscopy, abdominal CT or MRI were performed. SICUS examinations and ileo-colonoscopy were performed by experienced gastroenterologists aware of the diagnosis and clinical data, including bowel surgery, but each blinded to the results of other investigations and previous SICUS examinations.

### Small intestine contrast ultrasonography (SICUS)

Real-time ultrasound (US) was performed using Toshiba Tosbee (Tokyo, Japan) equipment with a 3.5 MHz convex and a 5 MHz linear array transducers. The apparatus can detect a bowel wall thickness (BWT) variation of 0.1 mm. Sonologist (NP) has performed more than 9,000 SICUS examinations.

**Figure 1:**
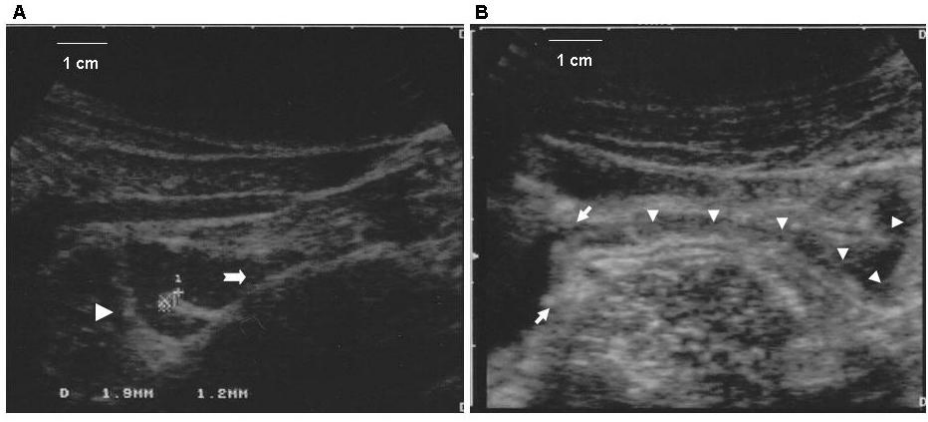
**A. SICUS assessment of ileo-colonic anastomosis**. Calipers indicate thickness of ileal (up) (1.9 mm) and colonic (down) (1.2 mm) limbs. Arrowhead indicates colon, arrow indicates neo-terminal ileum. **B. SICUS assessment of ileo-colonic anastomosis and neo-terminal ileum**. CD intramural involvement of ileo-colonic anastomosis (thickness 10 mm see arrows) and neo-terminal ileum (extending for 8 cm, see arrowhead).

SICUS was performed after an overnight fasting according to a previously published [4] method. Briefly, after the ingestion of 375 ml of macrogol contrast oral solution and after the contrast was seen to flow through the neo-terminal ileum into the colon, a retrograde follow-through assessment of the entire small bowel was performed visualizing, in a caudo-cranial sequence, the contrast-filled ileal and jejunal loops. The body position of patients was changed and abdominal compression with the ultrasound (US) transducer was used, whenever required, to improve visualization of any single loop and detection of intestinal abnormalities after the ingestion of the oral contrast. Wall thickness and lumen diameter were measured at several sites (proximal, middle and distal) of the small bowel at the level of maximally distended, and not contracting, intestinal loops. Wall thickness of the ileo-colonic anastomosis (ICA) including the ileal and colonic limbs (figure 1A) was calculated as the average of at least 3 measurements. Each ileal and colonic limb were separately measured from the mucosa to the outer rim of the muscularis propria. Maximal lumen diameter at the passage of the oral contrast at the level of ileo-colonic anastomosis was measured.

US criteria for recurrence of CD in the neo-terminal ileum was increased wall thickness (> 3 mm). The extension of the increased intestinal wall thickness (> 3 mm) (figure 1B) was reported as the average of at least 3 measurements.

Bowel stenosis was defined as lumen diameter less than 1 cm [5] measured at the level of neo-terminal ileum and/or ileo-colonic anastomosis. Bowel dilatation, defined as lumen diameter >2.5 cm [6,7], was also looked for.

At the end of the US investigation measurements of intestinal wall and lumen alterations at the level of ileo-colonic anastomosis and small bowel were reported on a standardized form.

### Ileocolonoscopy

Ileo-colonoscopy was performed by experienced gastroenterologists (MG, AG, FB, MT, AC) with experience of more than 15,000 examinations. Unless hampered by luminal stricture, the neo-terminal ileum was examined for 30 cm above the anastomosis. Multiple mucosal biopsies of neo-terminal ileum and the anastomotic large bowel were obtained in endoscopically normal mucosa as well as in the reddened mucosa in the proximity of epithelial lesions. The anastomosis was carefully inspected. At the end of the endoscopy, presence of mucosal lesions and grading according to Rutgeerts score [1] were reported on a standardized form. Ileal lesions were scored at ileocolonscopy as follows: 0, no lesions; 1, ≤5 aphtous lesions; 2, > 5 aphtous lesions with normal mucosa between the lesions or skip areas of larger lesions or lesions confined to the ileo-colonic anastomosis (i.e. < 1 cm in length); 3, diffuse aphtous ileitis with diffusely inflamed mucosa; 4, diffuse inflammation with already larger ulcers, nodules, and/or narrowing. Extension of mucosal lesions was also reported.

### Statistical analysis

In the analysis only pairs of SICUS and ileo-colonoscopy performed 2 weeks apart were included. To provide a descriptive comparison of patients and of US values, i.e. wall thickness at the level of ileo-colonic anastomosis and intramural lesions (wall thickness > 3 mm) at the level of neo-terminal ileum, arithmetic mean, standard deviation, median values and interquartile ranges were calculated. Box-plots [10] were used to provide an immediate graphical evaluation of US values by Rutgeerts score.

To our knowledge the normal wall thickness of the ICA has never been reported. Based on preliminary analysis an ICA wall thickness equal or more than 3.5 mm showed to better discriminate patients without, from those with, endoscopic lesion (score 1-4). Thereafter ICA wall thickness was dichotomised in the two categories ≤ 3.5 mm and > 3.5 mm, and endoscopic score in the two categories 0 and 1-4. Spearman correlation test was used to assess the relation between wall thickness values at the level of ileo-colonic anastomosis and extension of neo-terminal intramural lesions as measured by SICUS.

Univariate ordinal logistic regression models were applied to evaluate whether there was an association of SICUS values, age, gender, presence of symptoms, CDAI (< 150 or ≥150), CD duration before surgery, time interval between surgery and SICUS evaluation, with the different endoscopic scores. In order to assess the association of several characteristics in patients with less severe scores, patients with score ≥2 were grouped together. We further performed two multiple logistic regression analyses with outcomes score 0 vs > 0, and score 0 vs 1 (i.e., restricted only to Rutgeerts endoscopic score < 2), obtained through a backward selection strategy having excluded factors with a p-value > 0.20 obtained by a likelihood-ratio test. From these two logistic models, receiver operating characteristic (ROC) curves were then constructed to quantify the accuracy of combined measures of wall thickness of ileo-colonic anastomosis and extension of neo-terminal intramural lesions in discriminating score 0 from score 1-4, and score 0 from score 1. Finally, an ordinal logistic regression model was fitted including as covariates wall thickness of ileo-colonic anastomosis and extension of neo-terminal intramural lesions. The results from this model were then used to provide prediction of the risk of being classified in each of the three categorical endoscopic score (0, 1, and ≥2) for any given combination values of these two variables.

All the standard errors of the estimated parameters obtained by the previously described analyses were adjusted to take into account that the repeated measurements at different times for the same patient were clustered within the patient [11]. Two-sided p-values were defined statistically significant when *P *< 0.05, and marginally significant when 0.05 <*P *< 0.2. All the analyses were performed using STATA release 8.0 [12].

## Results

A complete SICUS investigation was performed in all evaluated patients after the ingestion of 375 mL of macrogol solution and none experienced any adverse event.

According to the study protocol the first observation was performed at 6 months after surgery in 19 patients (33%), at 12 months in 16 (28%), at 18 months in 5 (9%), and at 24 months in 18 patients (31%). Since not all patients underwent colonoscopy at the scheduled time, 18/58 patients performed one SICUS-ileocolonoscopy paired evaluation during a follow-up period of 35 ± 33 months (range 6-100), 25/58 two evaluations during a follow-up period of 44.6 ± 24.3 months (range 12-99), 13/58 three evaluations during a follow-up period of 59 ± 20.5 months (range 32-94) and 1 patient four evaluations during a follow-up period of 56 months for a total of 111 paired evaluations.

At 6 months after surgery, 58% of evaluated patients did not have recurrence (i.e. score 0), 31% had score 1 and 11% score ≥2. At 12 months after surgery 38% of evaluated patients did not have recurrence (i.e. score 0), 38% had score 1 and 24% score ≥2. At 24 months after surgery 6% of evaluated patients did not have recurrence (i.e. score 0), 22% had score 1 and 72% score ≥2.

### US and endoscopic findings

The main demographic and clinical characteristics and ileocolonoscopy of study population are reported in table 1. The total of 111 paired SICUS and ileo-colonoscopy observations performed 2 weeks apart were included in the analysis.

**Table 1:** Main demographic, endoscopic at first assessment and clinical characteristics of investigated subjects according to Montreal classification

Gender (M/F)	37/21
Median age (range years)	45.4 yrs (19-75 yrs)

Duration of Crohn's disease before surgery	44 ± 55 months

Illness behaviour at diagnosis (%)	
B1	4 (7%)
B2	32 (55%)
B3	22 (38%)

Location (%)	
L1	28 (48%)
L3	26 (45%)
L3+L4	4 (7%)

Previous surgical resection (%)	
2	11 (19%)
3	2 (3%)

Type of anastomosis	
Side to side	42 (72%)
End to side	11 (19%)
End to end	5 (9%)

Pathological findings at resection margins	
Negative	35 (60%)
Positive	6 (11%)
Not-reported	17 (29%)

Post-operative treatment	
5-ASA	44 (75%)
Azathioprine	14 (25%)

Rutgeerts score at first assessment (%)	
Score 0	20 (34%)
Score 1	15 (26%)
Score 2	7 (12%)
Score 3	5 (9%)
Score 4	11 (19%)

**Figure 2:**
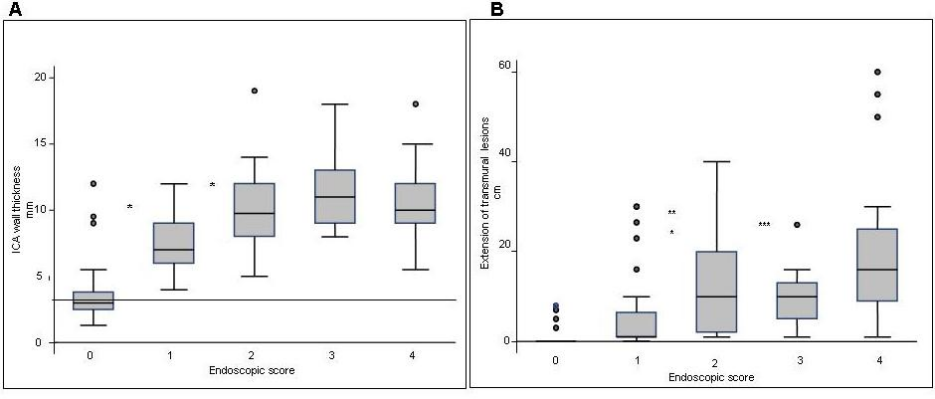
**A. Box-and-whiskers plots of wall thickness at the level of ileo-colonic anastomosis according to different Rutgeerts score**. The boxes at each score extend from the 25^th ^percentile (x_[25]_) to the 75^th ^percentile (x_[75]_) [i.e., the interquartile range (IQ)]; the lines inside the boxes represent the median values. The line emerging from the boxes (i.e., the "whiskers") extend to the upper and lower adjacent values. The upper adjacent value is defined as the largest data point ≤x_[75] _+ 1.5 × IQ, and the lower adjacent value is defined as the smallest data point ≥x_[25] _- 1.5 × IQ. Observed values more extreme than the adjacent values, if any, are individually plotted (circles). * p < 0.001 score 1 vs score 0, 2-4. The horizontal line indicates the cut-off value of 3.5 mm. **B. Box-and-whiskers plots of extension of intramural lesion at the level of neo-terminal ileum according different Rutgeerts score**. The boxes at each time unit extend from the 25^th ^percentile (x_[25]_) to the 75^th ^percentile (x_[75]_) [i.e., the interquartile range (IQ)]; the lines inside the boxes represent the median values. The line emerging from the boxes (i.e., the "whiskers") extend to the upper and lower adjacent values. The upper adjacent value is defined as the largest data point ≤x_[75] _+ 1.5 × IQ, and the lower adjacent value is defined as the smallest data point ≥x_[25] _- 1.5 × IQ. Observed values more extreme than the adjacent values, if any, are individually plotted (circles). * p < 0.001 score 1 vs score 4, ** p = 0.03 score 1 vs score 2, *** p = 0.04 score 1 vs score 3

Endoscopic score was 0 in 29 observations, 1 in 31, 2 in 18, 3 in 10 and 4 in 23. At SICUS, wall thickness of ileo-colonic anastomosis was 3 mm (median, IQR 2.5-3.8 mm) in patients with endoscopic score 0. It was 7 mm (median, IQR 6-9 mm) in patients with score 1. It was 9.8 mm (median, IQR 8-12 mm) in patients with score 2. It was 11 mm (median, IQR 9-13 mm) in patients with score 3, and 10 mm (median, IQR 9-12 mm) in those with score 4. There was a significant association between score 0/score 1-4 and wall thickness at the level of ileo-colonic anastomosis less or equal to 3.5 mm compared to more than 3.5 mm (p < 0.001). At the level of ileo-colonic anastomosis a wall thickness of more than 3.5 mm identified 100% of the patients with endoscopic score of 1-4 (figure 2A).

Wall thickness of ileo-colonic anastomosis was significantly greater in patients with Rutgeerts score 4, 3 and 2 compared to 1 (p < 0.001 for all comparisons) and in patients with score 1 compared to 0 (p < 0.001). Wall thickness of ileo-colonic anastomosis did not significantly differ among patients with score 2, 3 and 4 (figure 2A).

At SICUS the extension of intramural lesions (wall thickness > 3 mm) in the neo-terminal ileum was 1 cm (median, IQR 1-6.5 cm), 10 cm (median, IQR 2-20 cm), 10 cm (median, IQR 5-13 cm), and 16 cm (median, IQR 9-25 cm) in patients with score 1, 2, 3 and 4, respectively (figure 2B). The extension of intramural lesions at the level of neo-terminal ileum was significantly greater in patients with Rutgeerts score 4 (p < 0.001), 3 (p = 0.03) and 2 (p = 0.04) compared to score 1 and did not significantly differ among patients with score 2, 3 and 4 (figure 2B).

In 6 patients with early recurrence at 6 months after surgery endoscopic severity score was 1 and at SICUS wall thickness of the ileo-colonic anastomosis was 5-10 mm. In 4 patients with score 0 at ileocolonoscopy, Crohn's disease recurrent lesions, confirmed by MRI, were detected at SICUS as wall thickness of ileo-colonic anastomosis ranging between 5 mm and 12 mm (figure 2A) and as ileal-side extension of intramural lesion ranging between 3 cm and 8 cm (figure 2B). Histological inflammation was present in the biopsy of ileo-colonic anastomosis and neo-terminal ileum in all 4 patients.

**Figure 3:**
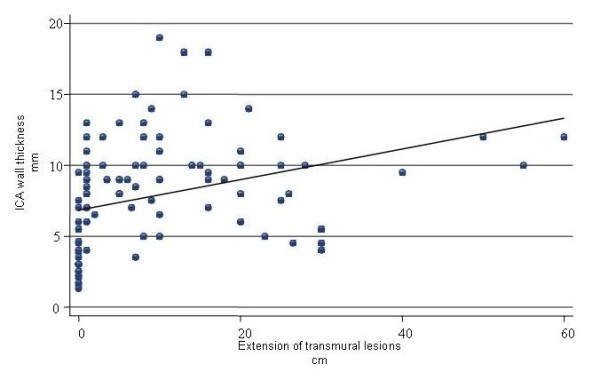
**Scatter plot of pairs of US values of the extension of intramural lesions at the level of the neo-terminal ileum and the wall thickness at level of the ileo-colonic anastomosis in 111 evaluations (58 patients) and estimated correlation**.

In patients with score 1, CD lesions were confined at ileo-colonic anastomosis in 29/31 (93%) patients at endoscopy and in 17/31 (54%) patients at SICUS. In 18/23 (78%) patients with score 4 the presence of stricture of ileo-colonic anastomosis did not enable to assess at endoscopy the pre-anastomotic ileal mucosa. In the remaining 5 patients the extension of mucosal lesion at endoscopy was significantly less than the extension of intramural lesion at SICUS (11 ± 3 cm vs 23 ± 16 cm). At SICUS a stenosis was confined at ileo-colonic anastomosis in 8 patients, its length was > 5 cm in 9 patients, and 3 cm in 1. There was a fair correlation (r = 0.467, p < 0.001) between wall thickness at the level of the ileo-colonic anastomosis and extension of neo-terminal intramural lesions as assessed at SICUS (figure 3).

### Association of demographic, clinical and US values with Rutgeerts severity score

Table 2 shows the association between demographic, clinical and US characteristics by the Rutgeerts severity score (i.e., 0, 1, and ≥2). At univariate analysis, the wall thickness value at the level of ileo-colonic anastomosis (ICA), the extension of intramural lesions at the level of neo-terminal ileum, age, and post-operative CD activity index (CDAI) at referral, were all significantly associated with Rutgeerts severity score. The older age and CDAI ≥150, while neither the disease duration before surgery, nor symptoms at referral, were associated with high grade severity of Rutgeerts score. Performing two multiple logistic regressions with outcome 0 vs > 1 - 4 and 0 vs 1, after a backward selection, only ileo-colonic wall thickness and extension of neo-terminal ileum intramural lesions were significantly associated with Rutgeerts score (Table 3).

**Table 2:** Demographic, clinical and SICUS characteristics of patients by Rutgeerts score at each pair evaluation (N = 111)

	Score 0	Score 1	Score ≥2	p value
Female, N (%)	11 (38%)	12 (39%)	15 (29%)	0.47

Male, N (%)	18 (62%)	19 (61%)	36 (71%)	

Median age, (IQR) yrs	34.7 (28.7-45)	43.4 (34.8-51)	53.9 (34.7-62)	0.01

Median CD duration before surgery (IQR) months	48 (7-63)	22 (1-68)	12 (3-120)	0.45

Median interval from surgery and evaluation (IQR) months	33.3 (11-59)	36 (20-66)	59.6 (25-114)	0.05

Median CDAI (IQR)	66 (29-93)	39 (17-94)	44 (23-144)	0.19

CDAI ≥ 150, N (%)	2 (7%)	2 (6%)	11 (22%)	0.04

CDAI < 150 N (%)	27 (93%)	29 (94%)	40 (78%)	

Presence of symptoms N (%)	9 (31%)	12 (39%)	29 (57%)	0.11

Median ICA wall thickness (IQR) mm	3 (2.5-3.8)	7 (6-9)	10 (9-12)	< 0.001

Median neo-terminal ileal extension of increased wall thickness, (IQR) cm	0	1 (1-6.5)	13 (7-20)	< 0.001

IQR: Interquartile range

**Table 3:** Estimated adjusted odds ratios (AOR) with 95% confidence intervals (95% CI) from two logistic models of having Rutgeerts score 0 vs 1-4 (section A), and 0 vs 1 (section B) on the basis of ICA wall thickness value and extension of increased (> 3 mm) neo-terminal ileal wall thickness

	AOR	95% CI	p value
**Score 0 vs. 1-4 (section A)**			

ICA wall thickness (for 1 mm increase)	1.96	1.22-3.15	0.01

Extension of increased (> 3 mm) neo-terminal ileal wall thickness (for 1 cm increase)	1.18	1.08-1.30	< 0.01

**Score 0 vs. 1 (section B)**			

ICA wall thickness (for 1 mm increase)	1.81	1.12-2.93	0.02

Extension of increased (> 3 mm) neo-terminal ileal wall thickness (for 1 cm increase)	1.15	1.06-1.24	< 0.01

It is of note that after adjusting for US variables, no statistically significant relationship was found with age and CDAI ≥150 when ICA wall thickness and extension of neo-terminal intramural lesions were entered in these models.

**Figure 4:**
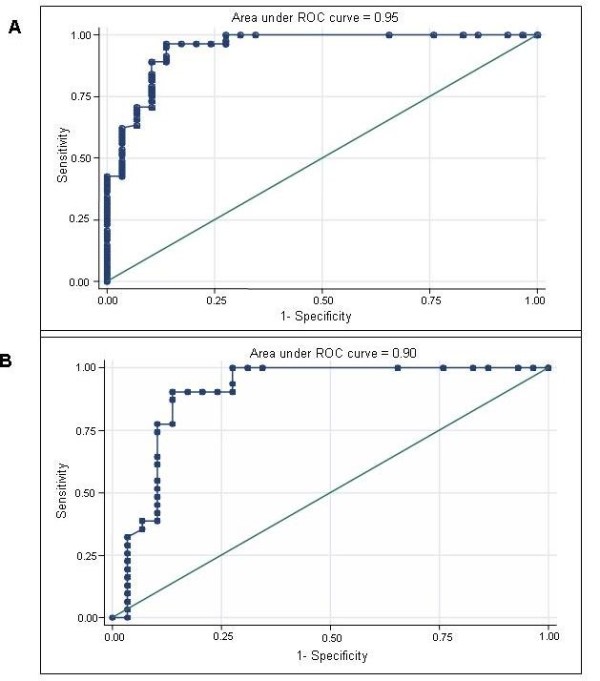
**ROC curve analysis**. Accuracy of the combined US values, i.e. intramural lesions extension at the level of the neo-terminal ileum and wall thickness values at the level of the ileo-colonic anastomosis, in discriminating patients with Rutgeerts score 0 vs 1-4 (section A) and 0 vs 1 (section B).

The ROC curve analysis combining the wall thickness of the ileo-colonic anastomosis with the extension of neo-terminal ileum intramural lesions shows a value of 0.95 (i.e., very good, given that 1 means perfect discrimination) in discriminating patients with score 0 from 1-4 and a value of 0.90 (i.e. good) in discriminating patients with score 0 from 1 (figure 4A and 4B).

**Figure 5:**
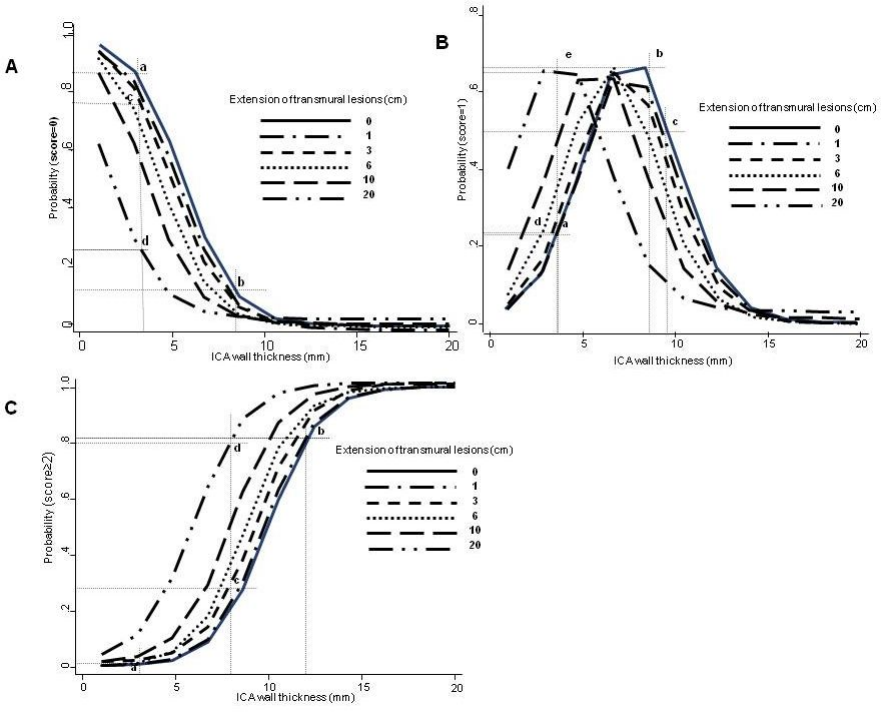
**Predicted probabilities of having a score of 0 (section A), 1 (section B), and ≥2 (section C) from a polychotomous ordinal logistic model with ICA wall thickness and extension of neo-terminal intramural lesions as covariates**. **Section A**. In absence of intramural lesion (extension 0) of neo-terminal ileum, the predicted probability to have score 0 is > 80% (**a**) when ICA wall thickness is ≤ 3.5 mm and progressively decreases to < 15% for ICA wall thickness ≥ 8 mm (**b**). The probability to have score 0 progressively decreases from 75% (**c**) to 23% (**d**) for intramural lesions of the neo-terminal ileum increasing from 3 cm to 20 cm. **Section B**. In absence of intramural lesion (extension 0) of neo-terminal ileum, the predicted probability to have score 1, progressively increases from 23% (**a**) to 66% (**b**) for wall thickness of ICA increasing from 3.5 mm to 8 mm. In absence of intramural lesion (extension 0) of neo-terminal ileum, the probability to have score 1 with ICA wall thickness > 9 mm is low (< 50%) (**c**). When the extension of intramural lesions at the level of neo-terminal ileum increases from 3 to 20 cm, the probability to have score 1 progressively increases from 24% (**d**) to 66% (**e**). **Section C**. In absence of intramural lesion (extension 0) of neo-terminal ileum, the predicted probability to have score ≥2 is < 1% (**a**) when ICA wall thickness is ≤ 3.5 mm and progressively increases to >80% (**b**) for ICA wall thickness >10 mm. With ICA wall thickness ≥8 mm and with intramural lesions of the neo-terminal ileum increasing from 3 cm to 20 cm, the probability to have score ≥2 progressively increases from 27% (**c**) to 80% (**d**).

The ordinal polychotomous logistic model analysis obtained after a backward selection starting from all variables investigated (see Table 2), in which the outcome was the Rutgeerts score severity (i.e., 0, 1, ≥2), showed again that only the US variables were associated with score severity (ICA wall thickness, for 1 mm increase: coefficient 0.73, 95%, CI 0.41-1.05, p < 0.001; extension of increased neo-terminal ileal wall thickness, for 1 cm increase: coefficient 0.14, 95% CI 0.08-0.21, p < 0.001). After adjusting for US variables the duration of disease before surgery was marginally associated with Rutgeerts score severity (coefficient 0.01, 95% CI 0.001-0.021 per one month increase, p = 0.07) (data not included in the table). Estimated risks of having/not having CD recurrence after surgery, taking into account both wall thickness at the level of ileo-colonic anastomosis and extension of neo-terminal ileum intramural lesions are shown in figure 5A-C. The predicted probability to have score 0, i.e. no recurrence, when ICA wall thickness is ≤ 3.5 mm, is > 80% in absence of intramural lesions of neo-terminal ileum (wall thickness ≤ 3 mm, i.e. extension 0) and it progressively decreases to 23% as intramural lesions of the neo-terminal ileum increases up to 20 cm. Conversely, the predicted probability to have score ≥ 2 is < 1% in absence of intramural lesions of neo-terminal ileum when ICA wall thickness is ≤ 3.5 mm and progressively increases to > 80% for ICA wall thickness ≥10 mm. The probability to have score ≥ 2 progressively increases from 27% to 80% as intramural lesions extension of the neo-terminal ileum increases from 3 cm to 20 cm when ICA wall thickness is ≥8 mm.

All analyses were performed also using only the first assessment, ultrasonography and endoscopy, for the 58 patients and the results were consistent with those presented (data not shown).

## Discussion

In patients with Crohn's disease (CD) submitted to ileal resection the evolution of post-operative recurrent CD lesions can be prospectively assessed since their onset. Patients with diffuse recurrent lesions in the neo-terminal ileum within 1 year of resection present symptoms earlier and are more prone to have complications than patients with no or very mild lesions. However, even minimal recurrent CD lesions such as aphtae have the tendency to progress into more severe involvement such as ulcerations and strictures [13]. Based on these observations it has been proposed that patients with CD have endoscopic evaluation of the neo-terminal ileum 6 to 12 months after surgery to guide therapeutic management [14]. Because of its invasiveness and need of intestinal preparation patients' compliance to undergo colonoscopy is poor [3]. Furthermore the endoscopy cannot explore the neo-terminal ileum in patients with score 4 and stricture of ileo-colonic anastomosis or of neo-terminal ileum. Finally ileo-colonoscopy cannot exclude other possible localizations in more proximal parts of small bowel requiring additional *ad hoc *investigations, the most widely used of which is small bowel standard radiology. However, because of radiation exposure radiology cannot be used at will and should be minimized [15] particularly in young people, child bearing age women, and in those who may require repetitive assessments for the follow-up of CD as in patients submitted to surgery.

A non-invasive method which visualizes the entire small bowel such as MRI [16-18] or US performed after the ingestion of oral contrast [4-7] is likely to improve patient's compliance to undergo follow-up controls that can be planned early after surgery and timely adjusted at will. A limitation of MRI is the inability to prolong the observation so to observe properly distended and not contracting small bowel loops even when performed with enteroclysis [16,17]. A limitation of oral contrast US could be the patient's refusal to ingest large amount of solution [8]. Following a previous SICUS study in which variable volumes (125 ml-1,000 ml) of macrogol solution were tested, the amount of 375 ml was identified as the one properly distending the entire small bowel [19] and acceptable by virtually all patients [6,7] as demonstrated in the present study.

Normal intestinal wall thickness of ≤3 mm has been identified at SICUS with repeated measurements at different levels of the small bowel in healthy subjects [5,20]. Based on this cut-off normal value of ≤3 mm it has been shown that the diagnostic accuracy of SICUS is comparable to that of small bowel follow-through [6,7] in detecting suspected small bowel pathology and of intraoperative findings in assessing site and extension of CD lesions [7].

Up to now the normal value of wall thickness of ICA in patients submitted to intestinal resection for CD complications is not known. In the present study the US value of 3.5 mm or more of wall thickness at the level of the ileo-colonic anastomosis predicted the presence of Crohn's disease recurrence in 100% of the patients. Increasing values of ICA wall thickness are significantly associated with increasing Rutgeerts severity score (figure 2A). Based on these findings the wall thickness of 3.5 mm appears to be the cut-off value to differentiate normal from abnormal ileo-colonic anastomosis in CD patients. To confirm this observation further studies should be performed in a larger CD population and comparatively in patients undergoing ileo-colonic anastomosis for non inflammatory disease.

Previous studies have assessed wall thickness after curative ileal resection in CD patients at the level of neo-terminal ileum with MR and US [17,20]. Both MR and US did not provide sufficient resolution to differentiate initial wall thickening in patients with endoscopic scores 1 and 2. Recently two ultrasound studies [8,9] reported that wall thickness >4 mm at the level of neo-terminal ileum had a high sensitivity in detecting severe endoscopic CD recurrence (i.e score 3 and score 4) as opposed to a low sensitivity in detecting mild lesions (score 1 and score 2). Differently from these studies limited to the US assessment of wall thickness of the neo-terminal ileum with no assessment of its extension, in the present one the wall thickness of the ileo-colonic anastomosis, expressed as the sum of the colonic and ileal limbs, and of the extension of intramural lesions of neo-terminal ileum were used in a model in order to differentiate patients with no recurrent CD lesions from those with recurrence and, more importantly, from those with low grade CD recurrence (i.e. score 1). The ROC curve analysis shows that the two combined variables represent an almost perfect tool in discriminating patients with score 0 from those with score 1-4 and a good tool in discriminating patients with score 0 from those with score 1. Therefore a relevant finding of the present study is that both variables, i.e., the increased wall thickness at level of the ileo-colonic anastomosis and the intramural extension of the lesion at the level of the neo-terminal ileum, should be taken into account to differentiate patients with endoscopic score 0 from score 1 and score 1 from score 2 and the latter from the more severe scores. At the first observation performed 6 months after surgery ICA wall thickening and normal wall thickness of the neo-terminal ileum was detected in 31% of the patients, indicating that a minimal involvement of the ICA precedes extension of the CD recurrent inflammation in the neo-terminal ileum. It is thus of note that assessment of ICA wall thickness at SICUS is relevant to detect postoperative recurrent lesion earlier than the usual US assessment of wall thickness limited to the neo-terminal ileum.

Differently from endoscopic scoring system, this study finds an association between the US grading of intramural lesion at the level of ICA and its extension along the neo-terminal ileum. The observation that in patients with Rutgeerts score 1 the lesions were confined to ICA in 17/31 (54%) at SICUS and in 29/31 (93%) at endoscopy suggests that a substantial intramural involvement may occur in the presence of few aphtae not accompanied by gross mucosal alterations. In addition, results of the present study indicate that while mucosal lesions were associated with intramural lesions in all patients with Rutgeerts score 1-4, in 4 patients intramural lesions, confirmed at MRI and histology, were not associated with mucosal lesions. This finding may indicate that CD inflammation does not necessarily imply the damage of the epithelial lining as also supported by the observation that in 3 patients with an early intramural recurrence at 6 months, mucosal lesions became apparent at ileo-colonoscopy 1 year later. Indeed it has been shown [21] that in the presence of inflammation, and occasionally even of granulomas at histology of the mucosal biopsy, endoscopic appearance can be completely normal. Thus recurrence can be detected as an intramural lesion without any accompanying mucosal alterations.

Similar to previous report [1], the degree of endoscopic severity of mucosal lesion in this study was not associated with the extension of wall thickening which was extremely variable, irrespective of the endoscopic score 1 (0-30 cm), 2 (0-40 cm), 3 (1-60 cm) and 4 (1-55 cm). Notably, in this study the presence of stenoses did not allow a proper assessment of CD recurrence at endoscopy in 78% of patients with score 4. In such circumstances, knowledge of the length of the stenosis, that can be assessed at SICUS and not at endoscopy, appears relevant for the possible indication to perform in these patients a perendoscopic dilatation. In the study by Rutgeerts et al patients with no (score 0) or very mild (score 1) and those with severe (score 3, and score 4) lesions at endoscopy were grouped together as they had, respectively, nearly asymptomatic or aggressive disease 1 year after surgery. Patients with intermediate severity of lesions (score 2) had no clear clinical prognosis as they progressed with either mild or aggressive disease.

By assessing both wall thickening at the level of the ileo-colonic anastomosis and its proximal extension in the neo-terminal ileum, SICUS could be proposed to grade the severity of intramural involvement of recurrent CD lesions in patients submitted to ileal resection. The proposed model predicts the absence of recurrence in more than 80%, and the presence of very mild CD recurrence (i.e. score 1) in more than 65%, of patients independently from any other variables.

Based on our results neither the CD disease activity as assessed with CDAI nor symptoms are associated with endoscopic score nor with severity and extension of intramural lesions, while the duration of CD before surgery appears to be marginally associated. This result may be explained by the low reliability of the CDAI system in the assessment of disease activity in patients with previous extensive ileo-colonic resection [22]. Identification of the minimal post-operative recurrence may be relevant in view of the development of more effective treatment that can be used to prevent the progressive development from mild to severe lesions that affects more than 70% of patients [13].

Since the US methods are operator-dependent the present study should have also assessed the inter-and intra-observer variability of SICUS findings. To limit intra-observer variability three measurements of ICA wall thickness and of the increased wall thickness extension in the neo-terminal ileum were performed and their mean calculated. Inter-observer variability should be evaluated in future studies.

## Conclusions

In conclusion the most relevant finding of the present study is that after ileal resection a non-invasive US method that evaluates the thickness of the ileo-colonic anastomosis, not differently from endoscopy, can detect initial, low grade CD recurrence, but, in addition and differently from endoscopy, it can assess the extension of the intramural lesion in all patients with CD recurrence even if luminal stenoses are present. A model based on the US assessment of the thickness of ileo-colonic anastomosis and the extension of the intramural involvement of the neo-terminal ileum may be proposed to grade the severity of the post-surgical recurrence in patients submitted to ileal resection for Crohn's disease.

## Competing interests

The authors declare that they have no competing interests.

## Authors' contributions

NP and EC contributed equally to this work; NP and EC designed research; PP planned and revised the statistical analysis. NP, MG, AG, FB, MT, AC performed SICUS and endoscopy. AM performed all histopathological analyses. NP and DP performed acquisition of data. NAH, GV and MDC participated to acquisition of data. PP and DP analyzed data; NP and EC wrote the paper.

All Authors read and approved the final manuscript

## Pre-publication history

The pre-publication history for this paper can be accessed here:

http://www.biomedcentral.com/1471-230X/10/69/prepub
